# Late Repair, One Year After a Knee Twisting Injury, of a Missed Femoral Trochlea Osteochondral Fragment, With Bioabsorbable Nails, in a 14-Year-Old Boy

**DOI:** 10.5435/JAAOSGlobal-D-18-00040

**Published:** 2019-08-05

**Authors:** Panos Megremis, Orestis Megremis, Rodanthi Margariti

**Affiliations:** From the A' Orthopaedic Department, Athens Children's Hospital “P. & A. Kyriakou”, Athens, Greece.

## Abstract

The authors report a case of a late repair of a missed, large, osteochondral fracture of the femoral trochlea in a 14-year-old boy due to lateral patellar dislocation after a twisting injury of the knee a year ago. The late—1 year after the knee injury—imaging assessment of the patient regarding radiograph images, CT scan, and MRI was misleading, misinterpreted, and failed to reveal this osteochondral fracture. The free osteochondral fragment was detected during diagnostic arthroscopy. Open reduction and fixation of the osteochondral fragment with bioabsorbable pins were done, and healing was achieved within an acceptable time. The patient's clinical and imaging examination with knee MRI, a year after the surgical treatment, was highly satisfactory. Fixation with bioabsorbable pins showed to be a worthwhile option in this case. The technique used was straightforward, with excellent short- and long-term results. Bioabsorbable pins may be used to restore successfully even an old, large osteochondral fracture in the immature skeleton.

Osteochondral fractures are more commonly associated with acute patellar dislocations and are frequently missed or misinterpreted in initial radiographs.^2,8,9^

The estimated yearly incidence of patellar dislocation in children is 43 per 100,000.^7^

Several authors have described osteochondral fractures after patellar dislocation, which commonly involve the patella and the lateral trochlear portions of the lateral femoral condyle.^2,3^

The articular cartilage has a limited ability to regenerate,^4^ and the articular surface begins to fill with the fibrocartilage within 10 days after the injury, so that the reduction of the osteochondral fracture becomes difficult and in some cases even impossible,^15^ making the early primary repair of large osteochondral fractures very important. The goal of the primary fixation of the osteochondral fragment is to restore the joint surface and prevent secondary osteoarthritis.

Although an osteochondral fracture should always be recognized immediately after the injury, the fracture often escapes detection, until the patient presents knee pain and intermittent episodes of knee swelling and blocking.

Treating displaced osteochondral fractures in growing children remains a challenge for the orthopaedic surgeon. Removal of a large loose bone fragment results in the early onset of osteoarthritis. This case report describes the late fixation of a large, intra-articular osteochondral fragment due to patellar dislocation with bioabsorbable pins a year after the injury, postoperative treatment, and follow-up results founded on subjective symptoms, clinical examination, and imaging examination, including MRI.

## Case Report

A 14-year-old boy presented to our clinic, complaining of mild right knee pain, which exacerbated in sports activities and intermittent episodes of knee swelling and blocking. He reported a foreign-body feeling in the knee.

No recent knee injury was stated, but he recalled a twisting injury of the right knee 1 year ago. After the injury, the right knee was swollen and painful, but the patient refused to seek any medical advice and treatment. He remained in bed for a few days only, and he gradually returned to his normal daily activities, avoiding participating in any sports since then.

At the present clinical examination, a moderate degree of right knee swelling with a painless, full range of knee motion was found.

**Figure 1:**
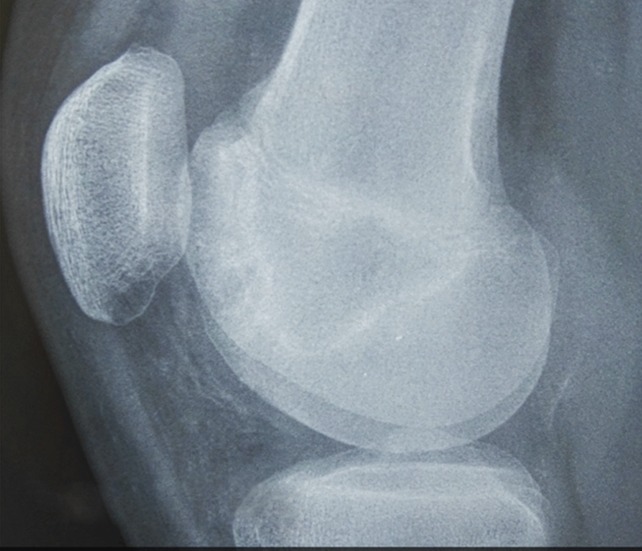
Right knee radiograph image. Lateral view. Not well-identifiable cystic lesions of the femoral trochlea.

**Figure 2:**
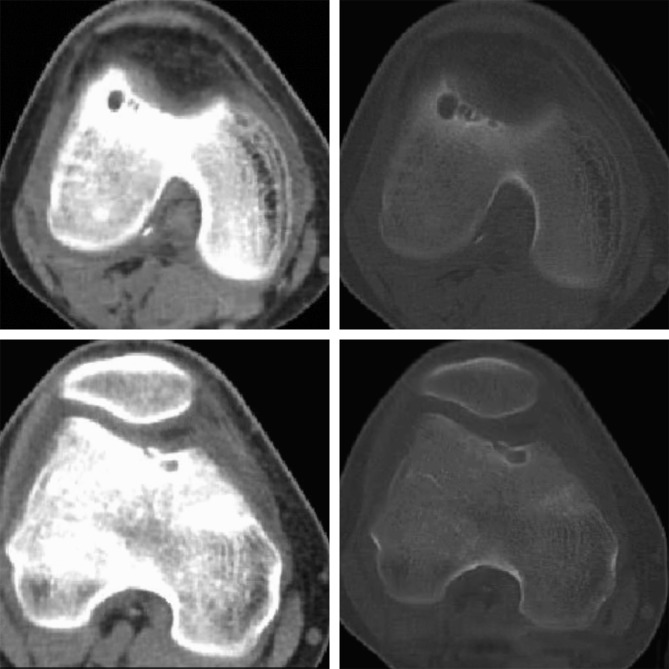
Knee CT scan image. Cystic lesions both in the lateral and medial sides of the femoral trochlea.

**Figure 3:**
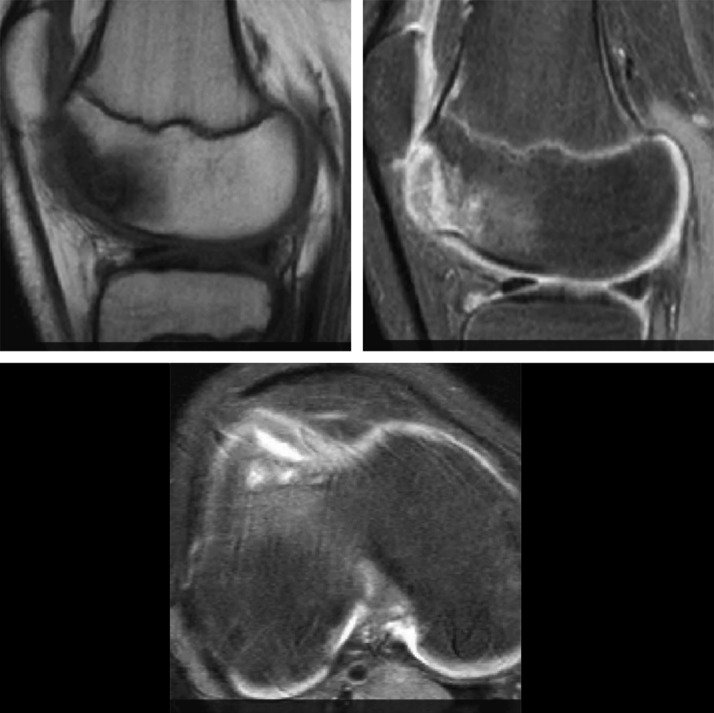
Knee MRI image. The low signal in the T1-weighted image and the high signal in the T2-weighted image.

The imaging assessment of the patient's right knee with radiograph images, CT scan, and MRI was misleading and misinterpreted. Anterior-posterior radiographs of the right knee were completely negative, whereas on the lateral radiographs, a suspicion of cystic lesions at the femoral trochlea was found (Figure 1). On the CT scan of the right knee, well-defined cystic lesions of different sizes in both the lateral and medial sides of the femoral trochlea were present (Figure 2). The MRI showed a low signal in T1-weighted images and a high signal in T2-weighted images at the femoral trochlea (Figure 3).

**Figure 4:**
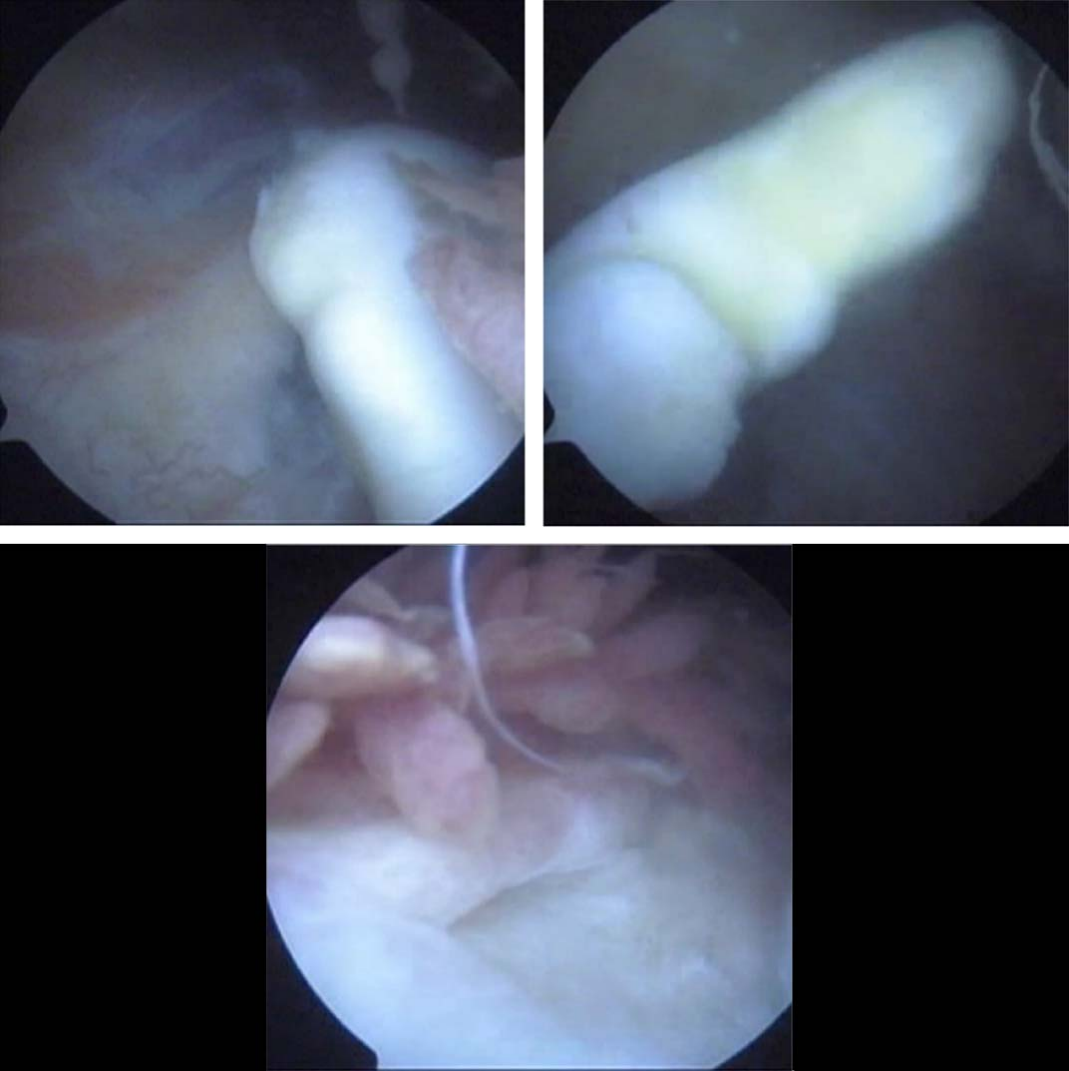
Knee arthroscopic view. A large osteochondral loose body, floating in the suprapatellar pouch. A large osteochondral defect of the femoral trochlea, filled with fibrous tissue.

Diagnostic arthroscopy of the right knee was decisive and revealed a large osteochondral loose body (>4 cm^2^), floating in the suprapatellar pouch, such as a large osteochondral defect of the femoral trochlea, filled with fibrous tissue (Figure 4). Furthermore, a small defect in the lateral facet of the patella was detected, which was already covered with fibrocartilaginous tissue. A free small osteochondral fragment (<1 cm^2^), found in the intercondylar notch, apparently detached from the patellar lateral facet, was removed. The lateral facet of the patella remained intact.

**Figure 5:**
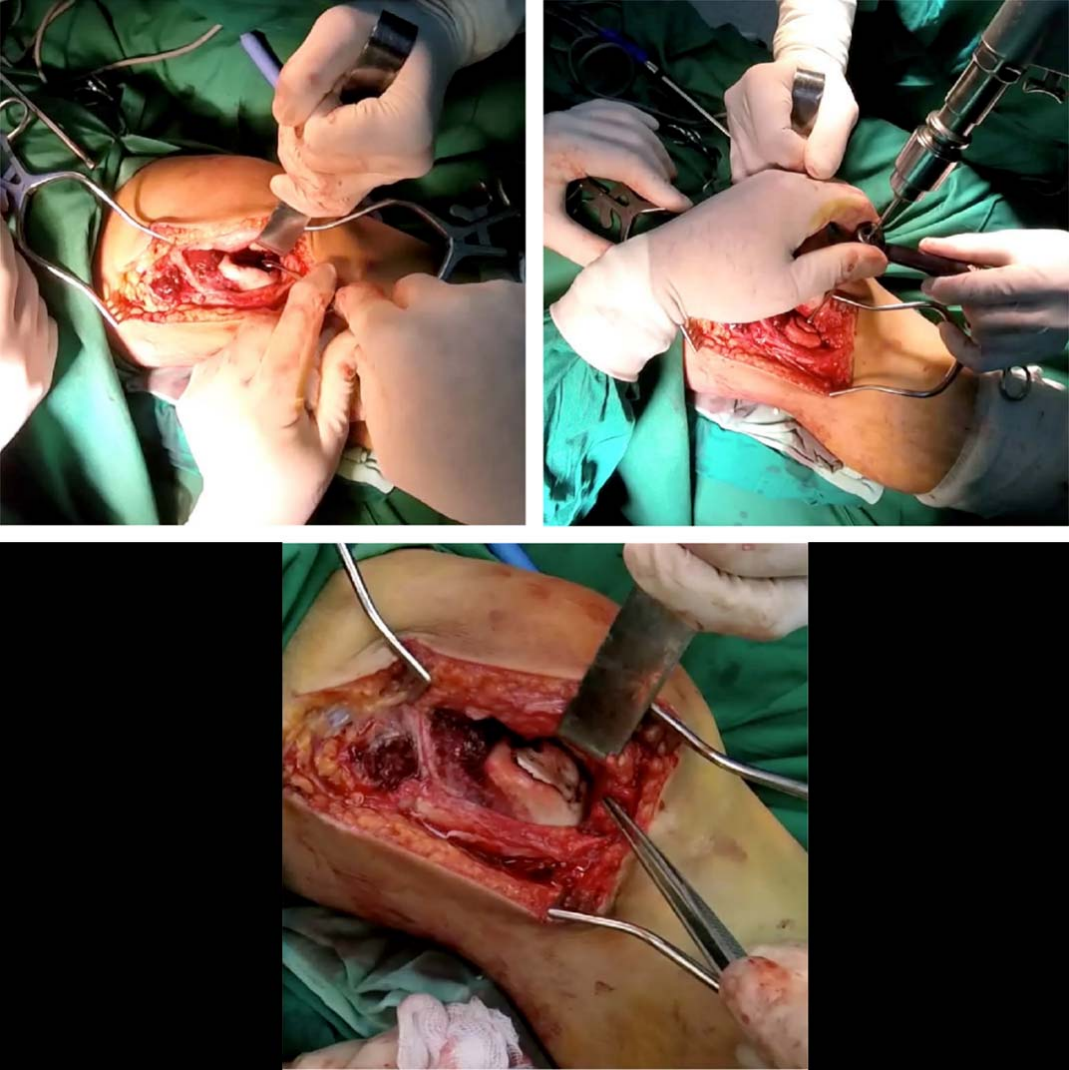
Débridement of the subchondral sclerotic bone of the femoral trochlea crater, with a spoon-shaped curet. Fixation of the osteochondral fragment with five, 1.5-mm bioabsorbable pins.

A lateral parapatellar skin incision was applied to achieve the open reduction of the free large osteochondral fragment onto the trochlear osteochondral defect. For the débridement of the subchondral sclerotic bone of the femoral trochlea crater (Figure 5), a spoon-shaped curet was used. Trimming of the free osteochondral fragment with a blade was necessary for the proper fitting of the osteochondral fragment into its position at the femoral trochlea defect. The fixation of the osteochondral fragment was done with five, 1.5-mm bioabsorbable pins (Figure 5) .

Postoperatively, the knee was immobilized in extension in a Scotchcast knee splint for 3 weeks and then in a functional knee orthosis, keeping the knee in extension, for another 3 weeks without weight-bearing. After this period of 6 weeks, partial weight-bearing started gradually and increased to full weight-bearing at the end of the eighth postoperative week. Simultaneously, the knee flexion gradually increased to full flexion within 4 weeks (till the end of the 10th postoperative week) with adjusted knee orthosis.

**Figure 6:**
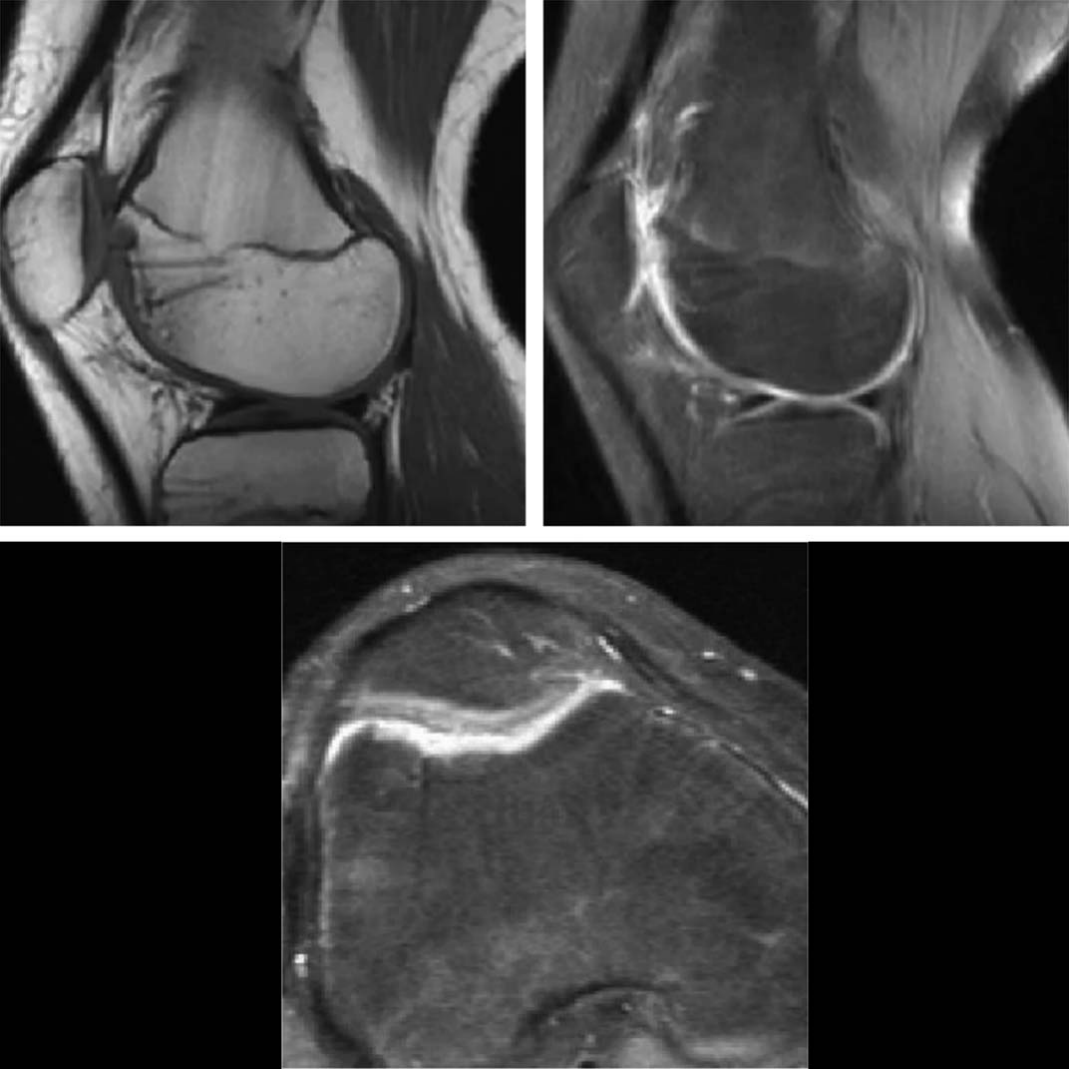
Knee MRI images 6 months postoperatively. Full consolidation of the osteochondral fragment in the T1- and T2-weighted images.

Knee MRI was done 6 months after surgery and showed full consolidation of the osteochondral fracture (Figure 6). In the last follow-up, 18 months postoperatively, the patient had returned to his previous sport activities without any restriction. The range of motion of the right knee was full, regarding the extension and flexion.

## Discussion

To the best of our knowledge, this is the first report to describe a successful late repair of an osteochondral fracture of the trochlea due to patellar dislocation, through internal fixation of the free osteochondral fragment with bioabsorbable pins a year after injury. The literature search led to only two studies: The first one reported about the repair of a patellar osteochondral fracture 8 weeks after trauma,^11^ and the second described the fixation of an osteochondral fracture of the lateral femoral condyle 3 months after the injury.^15^

In most cases of an osteochondral fracture diagnosed more than 15 days after injury, the detached fragment was classically excised.^13,14^ According to the size of the osteochondral defect, another surgical technique had to be chosen, to repair the defect of the articular cartilage to prevent secondary osteoarthritis. Marrow stimulation and resurfacing techniques, such as microfracture, autologous chondrocyte implantation, and mosaicplasty, showed to give encouraging clinical results, but they seem to present many limitations. Microfracture provides healing with mature fibrocollagen that contains predominantly type I collagen with only small amounts of type II collagen, resulting in a deficiency in durability compared with the articular cartilage. In fact, many authors describe a deterioration of the clinical condition over time, which results in restriction of the activity level of the patient.^4^ The mosaicplasty seems to be suitable only for small osteochondral defects because of the donor site morbidity, whereas it is considered to be contraindicated for patellar defects because of the widely different thicknesses of the donor and recipient articular cartilage.^1^ The long-term outcome for mosaicplasty seems to be doubtful.^1^ Autologous chondrocyte implantation is a technically complicated and time-consuming two-stage method, which needs to be more investigated regarding the technical details and long-term outcome.

The imaging assessment to diagnose an osteochondral fracture due to patellar dislocation includes usually plain radiographs (in anterior-posterior and lateral views), CT scan, which is helpful to determine the exact size and location of the osteochondral fragment as well as its origin, and MRI. The MRI is regarded as the benchmark to analyze the physics of the trauma and estimate possible concomitant injuries.^9,13^ In our case, the imaging assessment had limited efficacy in detecting the osteochondral fracture and the free osteochondral fragment. Owing to the unclear findings, diagnostic arthroscopy became necessary and showed the dimension of the injury. As part of the same surgical procedure, open reduction and fixation of the osteochondral fragment at the femoral trochlea with bioabsorbable pins were done. An open approach, medial, or lateral parapatellar for the reduction and fixation of the osteochondral fragment is a common procedure for large lesions involving a clinically important area of the weight-bearing articular surface.^11,13–15,19^

Fixation of large osteochondral fragments can be achieved with metal pins, allograft cortical bone pins, Herbert screws, absorbable sutures, meniscus arrows, and biodegradable screws and pins.^5,6,17,18^ In our case, bioabsorbable pins were applied to fixate the osteochondral fragment. This technique is advantageous because no additional surgery was needed to remove the implants.

According to the excellent outcome in this young patient, we may assume that no certain time limits to perform osteochondral fracture repair were found. Therefore, the primary fixation of an osteochondral fragment should be attempted even in missed cases. In our opinion, the only limit to be considered is the integrity of the osteochondral fragment to be fixated.
